# Modelling spatiotemporal dynamics of cerebral blood flow using multiple-timepoint arterial spin labelling MRI

**DOI:** 10.3389/fphys.2023.1142359

**Published:** 2023-05-26

**Authors:** Joana Pinto, Nicholas P. Blockley, James W. Harkin, Daniel P. Bulte

**Affiliations:** ^1^ Institute of Biomedical Engineering, Department of Engineering Science, University of Oxford, Oxford, United Kingdom; ^2^ David Greenfield Human Physiology Unit, School of Life Sciences, University of Nottingham, Nottingham, United Kingdom; ^3^ Poole Hospital NHS Foundation Trust, Poole, United Kingdom

**Keywords:** arterial spin labelling, cerebral blood flow, functional MRI, kinetic modelling, cerebral haemodynamic

## Abstract

**Introduction:** Cerebral blood flow (CBF) is an important physiological parameter that can be quantified non-invasively using arterial spin labelling (ASL) imaging. Although most ASL studies are based on single-timepoint strategies, multi-timepoint approaches (multiple-PLD) in combination with appropriate model fitting strategies may be beneficial not only to improve CBF quantification but also to retrieve other physiological information of interest.

**Methods:** In this work, we tested several kinetic models for the fitting of multiple-PLD pCASL data in a group of 10 healthy subjects. In particular, we extended the standard kinetic model by incorporating dispersion effects and the macrovascular contribution and assessed their individual and combined effect on CBF quantification. These assessments were performed using two pseudo-continuous ASL (pCASL) datasets acquired in the same subjects but during two conditions mimicking different CBF dynamics: normocapnia and hypercapnia (achieved through a CO_2_ stimulus).

**Results:** All kinetic models quantified and highlighted the different CBF spatiotemporal dynamics between the two conditions. Hypercapnia led to an increase in CBF whilst decreasing arterial transit time (ATT) and arterial blood volume (aBV). When comparing the different kinetic models, the incorporation of dispersion effects yielded a significant decrease in CBF (∼10–22%) and ATT (∼17–26%), whilst aBV (∼44–74%) increased, and this was observed in both conditions. The extended model that includes dispersion effects and the macrovascular component has been shown to provide the best fit to both datasets.

**Conclusion:** Our results support the use of extended models that include the macrovascular component and dispersion effects when modelling multiple-PLD pCASL data.

## 1 Introduction

Imaging studies quantifying cerebral blood flow (CBF) have been increasingly applied in an effort to characterize brain health and baseline CBF has been known to be an important physiological parameter that is commonly altered at earlier stages of several pathological conditions, including Alzheimer’s disease, stroke and small vessel disease (Alsop et al., 2015; Haller et al., 2016; De Vis et al., 2018; Lindner et al., 2023). CBF can be quantified non-invasively using the arterial spin labelling (ASL) MRI contrast, with most studies using single timepoint approaches (Alsop et al., 2015). However, in specific pathologies, as well as under certain physiological states where CBF dynamics are altered, single timepoint acquisition schemes and their assumptions might be invalid, ultimately compromising CBF quantification. One of those conditions is when acquiring data during a hypercapnia challenge, which is the case when evaluating cerebrovascular reactivity (CVR). CVR is the intrinsic mechanism of cerebral blood vessels of adjusting their calibre in response to a vasoactive stimulus. CVR has been shown to also be impaired in several pathologies, possibly providing additional or complementary information to baseline CBF (Catchlove et al., 2018; Chen, 2018). The most common way to evaluate CVR is by increasing arterial blood partial pressure of carbon dioxide (PaCO_2_) (Moreton et al., 2016; Pinto et al., 2021) and imaging the concomitant CBF changes using an appropriate modality such as ASL. In this case, CVR can be quantified as the change in CBF in response to a change in PaCO_2_ (Mandell et al., 2008; Sobczyk et al., 2015).

However, an increase in PaCO_2_ concentration is also expected to alter blood flow dynamics, with an increase in blood flow velocity and shortening of transit times (Donahue et al., 2016). This can potentially make approaches and assumptions commonly used for baseline CBF quantification inaccurate under these conditions (e.g., fixed transit time, unchanged bolus shape). This issue can be partially overcome by using an ASL multiple time-point acquisition strategy (multiple-PLD) and fitting this signal using an appropriate physiological model. This approach allows estimation of CBF as well as other related features, such as the time it takes for the labelled blood to flow from the labelling region to the vascular or tissue compartment of the imaging regions (arterial transit time, ATT) (Donahue et al., 2016; Zhao et al., 2021), or the volume of blood signal arising from larger arteries that is destined for more distal tissues (arterial blood volume, aBV) (Chappell et al., 2010). Additionally, most ASL studies assume that the shape of the labelled blood bolus remains unaltered during the transit time of the label through the vasculature. However, due to effects collectively known as dispersion and including different laminar flow profiles, vessel architecture, or diffusion of the labelled water, the bolus shape is in fact altered throughout the vascular tree (Wu et al., 2007; Gallichan and Jezzard, 2008; Kazan et al., 2009; Chappell et al., 2013). By correcting for this effect, as some dispersed spins might not have arrived at their final destination, CBF estimation can be improved while potentially also refining the separation between the aBV and tissue components (if these are modelled separately). The impact of modelling dispersion and aBV effects in ASL has been recently investigated during normocapnia (van der Plas et al., 2022), however, given the change in blood velocity and CBF temporal features that occurs during hypercapnia or in pathologies that alter CBF dynamics, the impact of these modelling strategies might be different. In this work, we test several modelling strategies that include dispersion and/or macrovascular contribution and assess their effect on the quantification of CBF spatiotemporal dynamics during two different physiological states, normocapnia and hypercapnia.

## 2 Materials and methods

### 2.1 Data acquisition

A group of 10 healthy subjects (5 M, 20.4 ± 0.8 years old) was studied on a 3 T Siemens Prisma Scanner with a 32 channel receive only head coil (Blockley et al., 2016). All participants provided written, informed consent in order to take part in the study and ethical approval was obtained from the Central University Research Ethics Committee (CUREC) at Oxford University.

Functional MR scanning included a multiple-PLD pseudocontinuous ASL (pCASL) sequence (Okell et al., 2013) with a 2D multi-slice GE-EPI readout, background suppression, and the following parameters: spatial resolution = 3.5 × 3.5 × 5 mm^3^, TR/TE = 4,100/14 ms, bolus duration = 1400 ms, 6 PLDs (250, 500, 750, 1,000, 1,250, and 1,500 ms), 8 averages for each PLD, number of slices = 24, time per slice = 46 ms and total acquisition time of 6 min and 40 s. Background suppression was achieved with a pre-saturation module (WET) and optimally timed global hyperbolic secant inversion pulses. An M_0_ calibration image with no labelling or background suppression was also collected. A field map was acquired using a 2D Fast Low Angle Shot (FLASH) method with the following parameters: TR 378 ms, TE1/TE2 4.92 ms/7.38 ms, FOV of 220 mm × 220 mm, matrix 64 × 64, slices 24, slice thickness 4.5 mm, slice gap 0.45 mm, flip angle 45°. A T_1_-weighted structural image was also acquired for each subject using a 3D Magnetisation Prepared Rapid Acquisition Gradient Echo (MPRAGE) pulse sequence with the following parameters: TR 1.9 s, TE 3.74 ms, FOV 174 mm × 192 mm × 192 mm, matrix 116 × 128 × 128, flip angle 8°, inversion time (TI) 904 ms.

**FIGURE 1:**
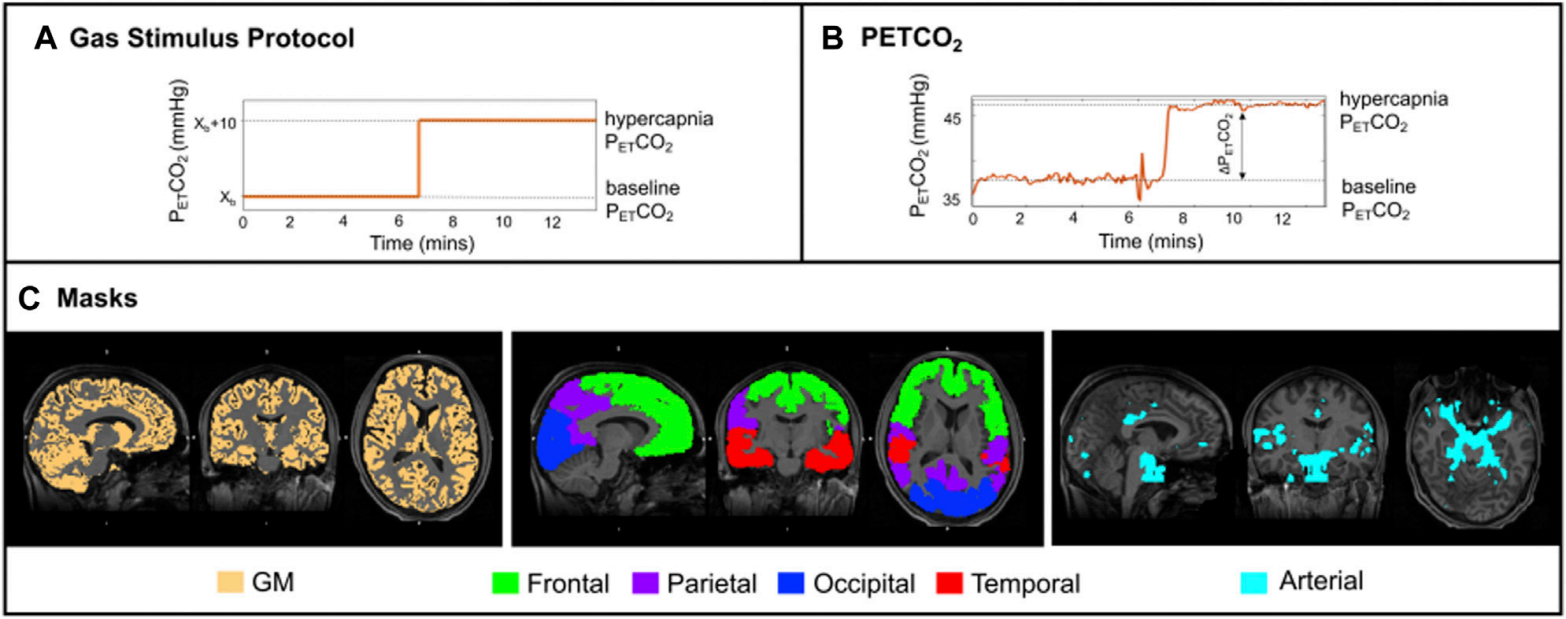
**(A)** Schematic of the stimulus paradigm; **(B)** PETCO_2_ trace of one illustrative subject. ASL data acquisition was performed only during the plateau periods; **(C)** Orthogonal representations, for one illustrative subject, overlaid on the structural image: (left) thresholded GM mask in yellow; (middle) four regions of interest (ROIs—frontal, parietal, occipital, and temporal lobes); (right) arterial mask in light blue.

The gas challenge was delivered by a computer controlled gas blender (RespirAct™ Gen 3, Thornhill Research Inc., Toronto, Canada) that implements a prospective algorithm for the targeting and maintenance of end-tidal CO_2_ partial pressure (PETCO_2_) and end-tidal O_2_ partial pressure (PETO_2_) concentrations (Slessarev et al., 2007). The gas protocol (Figure 1A) was personalised to each subject’s PETCO_2_ and PETO_2_ baseline values. Modulations in PETCO_2_ were targeted relative to baseline, whilst maintaining PETO_2_ constant (Figure 1B). Other details on the gas challenge setup can be found in (Blockley et al., 2017). The pCASL gas protocol consisted of a baseline period of normocapnia followed by a period of hypercapnia (PETCO_2_ step change of +10 mmHg). Both periods lasted 6 min and 40 s (Figure 1A).

### 2.2 Data analysis

Offline data processing was performed using FSL 6.0.3 [FMRIB Software Library (Jenkinson et al., 2012)], Matlab R2019b (Mathworks, Natick, MA, United States), and the IBM SPSS statistics tool (v.27).

Structural images were tissue segmented using FAST (Zhang et al., 2001), to produce grey matter (GM), white matter and cerebrospinal fluid partial volume estimate (PVE) maps. The GM PVE maps were further thresholded at 0.3, creating restrictive GM masks whilst maintaining a significant number of voxels within regions of interest (Figure 1C). Co-registration from functional to structural space was performed using a linear registration tool, FLIRT (BBR) (Jenkinson et al., 2002), and from structural to standard (MNI) space was done using FLIRT and a non-linear registration (FNIRT) tool (Andersson et al., 2007). These registrations were also used to transform four regions of the MNI structural atlas (frontal, parietal, temporal and occipital lobes, Figure 1C) (Mazziotta et al., 2001) and the segmented structural images to functional space. Individual arterial masks were also considered, and these were obtained by thresholding the arterial masks that resulted from model fitting (more details below).

The multiple-PLD pCASL datasets underwent standard pre-processing using FSL. Steps included extraction of first volume (M_0_), removing non-brain structures using BET (Smith, 2002), motion correction using MCFLIRT (Jenkinson et al., 2002), and distortion correction using a field-map strategy (FUGUE). Pairwise subtraction of label and control images was then performed in order to generate perfusion-weighted images (∆M).

Model fitting of the multiple-PLD pCASL data for parameter quantification was performed by applying a Bayesian approach with the default parameter prior information (BASIL, http://fsl.fmrib.ox.ac.uk/fsl/fslwiki/BASIL). Bayesian modelling strategies have been shown to provide robust and reliable results for ASL data quantification, by using prior knowledge based on physically realistic ranges of the parameters (Chappell et al., 2009). In particular, in this work we have modelled our ASL data using a standard kinetic model (Buxton et al., 1998), and incorporating other physiological contributions, creating extended models (Chappell et al., 2009). In particular, we have explored the impact of modelling the intravascular blood water that is destined to perfuse more distant tissues (also known as macrovascular or arterial component, aBV) using the model proposed by (Chappell et al., 2010) (aBV with an automatic relevancy determination prior and ATT prior with mean set at 1 and precision set at 1). Additionally, we also tested for the impact of modelling dispersion effects, using a gamma distribution shaped kernel as proposed by (Chappell et al., 2013), with parameters: time to peak (p) and sharpness (s) [parameters reparametrized and subject to a Gaussian prior with means described by log (s) = 2 and log (s*p) = −0.3 and precision set at 1]. All models are implemented in BASIL. A combination of different modelling strategies was used to assess the impact of these on parameter estimation when using different conditions (normocapnia and hypercapnia) (Table 1). Four different models were tested: 1) with arterial component but without dispersion effects (M_art_M_nodisp_), 2) with the arterial component and dispersion effects (M_art_M_disp_), 3) without arterial component and dispersion effects (M_noart_M_nodisp_), and 4) without arterial component but with dispersion effects (M_noart_M_disp_).

**TABLE 1:** The four different extended models including dispersion and/or macrovascular contributions.

**Model Options**			
		Dispersion	
		Yes	No
Macrovascular Component	Yes	M_art_M_disp_	M_art_M_nodisp_
	No	M_noart_M_disp_	M_noart_M_nodisp_

The resulting CBF and aBV maps were calibrated using a voxelwise approach within BASIL, assuming a labelling efficiency of 0.85 (Pinto et al., 2020). CVR was computed as CBF change due to hypercapnia normalized by the corresponding change in PETCO_2_.

Average parameter values were computed for the following regions intersected with the total GM mask: frontal, parietal, temporal, and occipital (Figure 1C, middle). A total GM mask was also considered (Figure 1C, left), as well as an arterial mask obtained by thresholding the corresponding arterial blood volume maps results from the model that includes dispersion and the arterial component, M_art_M_disp_ (visually optimised threshold of aBV > 0.7, Figures 1, 4). The Bayesian approach used in this study also allows for model comparisons to be performed through the estimation of the free energy (FE) (Chappell et al., 2009). FE approximates the Bayesian evidence for a model, and thus combines the accuracy of a model’s fit to the data with a penalty for the number of free parameters in the model. The closer FE is to zero the better the model is at explaining the data.

To evaluate differences between average parameters across regions, models, and conditions, a repeated-measures 3-way Analysis of Variance (rm-3-way-ANOVA, *p* < 0.05, Greenhouse-Geisser correction for sphericity), with factors: condition, region, and model, was applied. Post-hoc analysis was done using simple effects tests and pairwise comparisons with Bonferroni correction for multiple comparisons.

## 3 Results

Table 2 summarizes the main demographic descriptors of each subject as well as the corresponding ∆PETCO_2_ values acquired during the multiple-PLD pCASL acquisitions. The inhalation of a gas mixture with higher content of CO_2_ (hypercapnia) significantly altered individual PETCO_2_ values with an average increase of approximately 8 mmHg (*p* < 0.001).

**TABLE 2:** Demographic data and PETCO_2_ values for each subject. Bottom row corresponds to the mean and standard deviation (mean ± SD) across subjects. M stands for male and F for female.

Subject	Age	Sex	PETCO_2_ normocapnia	PETCO_2_ hypercapnia	∆PETCO_2_
1	21	M	38.0 ± 0.5	46.2 ± 0.4	8.2
2	21	M	37.8 ± 0.8	44.2 ± 0.4	6.4
3	21	M	42.1 ± 0.8	50.5 ± 0.4	8.4
4	19	M	42.6 ± 0.8	49.8 ± 0.3	7.2
5	20	F	39.3 ± 1.2	47.7 ± 0.5	8.4
6	21	F	33.0 ± 2.8	41.6 ± 0.9	8.6
7	21	F	38.7 ± 0.6	47.3 ± 0.3	8.7
8	21	M	39.9 ± 0.8	47.7 ± 0.3	7.8
9	19	F	38.4 ± 0.9	45.5 ± 1.2	7.1
10	20	F	38.2 ± 0.7	45.5 ± 2.0	7.3
mean ± SD	20.4 ± 0.8	5 F/5 M	38.8 ± 2.6	46.6 ± 2.6	7.8 ± 0.8

**FIGURE 2:**
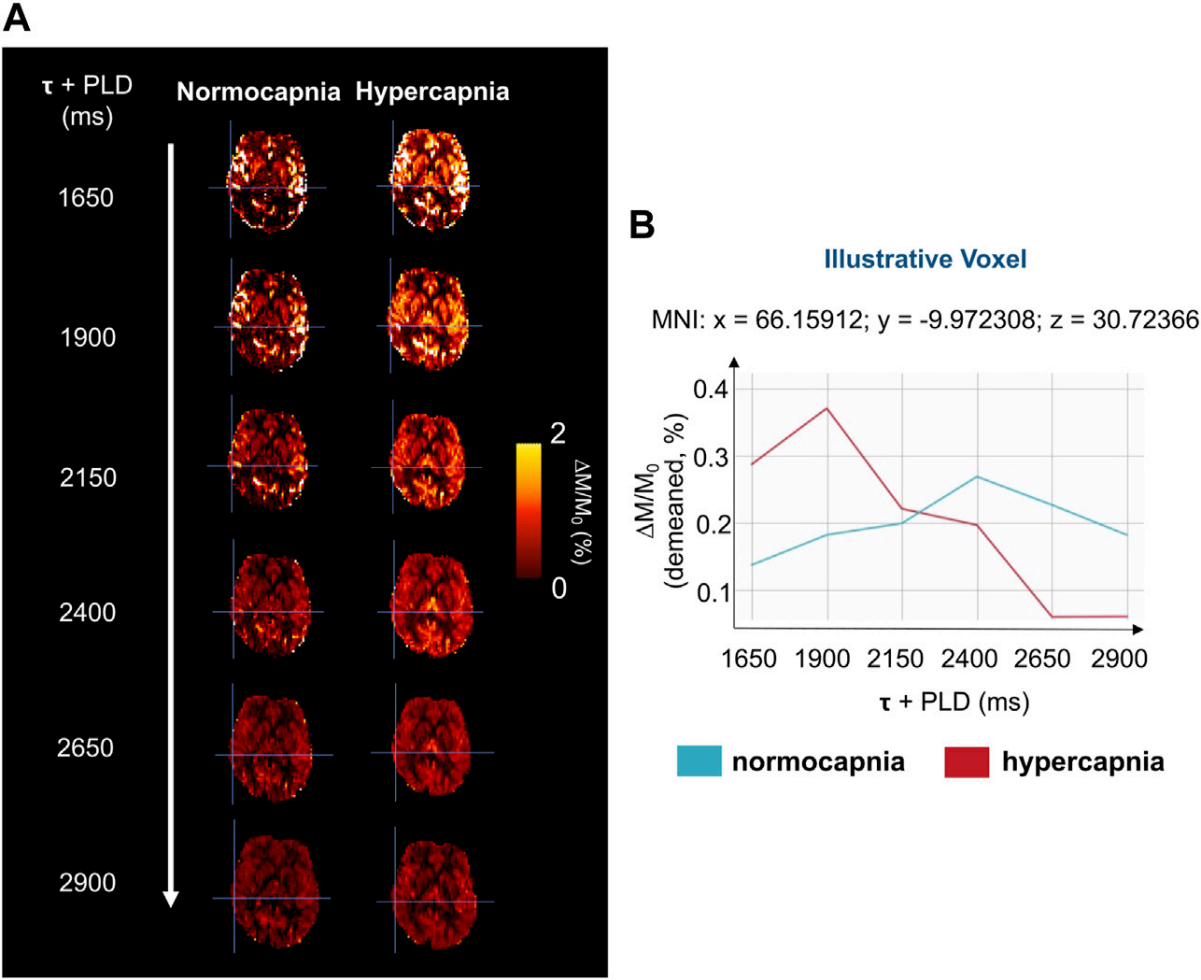
**(A)** Illustrative magnetization difference images (control-label, ΔM) normalized by corresponding calibration image (M_0_), of a representative subject and brain slice, throughout the different τ + PLDs. **(B)** Kinetic curves of a representative voxel (highlighted in Figure 2A), for the two gas challenges. The curves have been demeaned for clarity and better visualization.

Figure 2A shows illustrative images of the ASL difference normalized by the corresponding M_0_ image, ΔM/M_0_, across time (τ + PLDs) for the two conditions (normocapnia and hypercapnia). The corresponding kinetic curves for a representative voxel highlighted in Figure 2A can be seen in Figure 2B.

**FIGURE 3:**
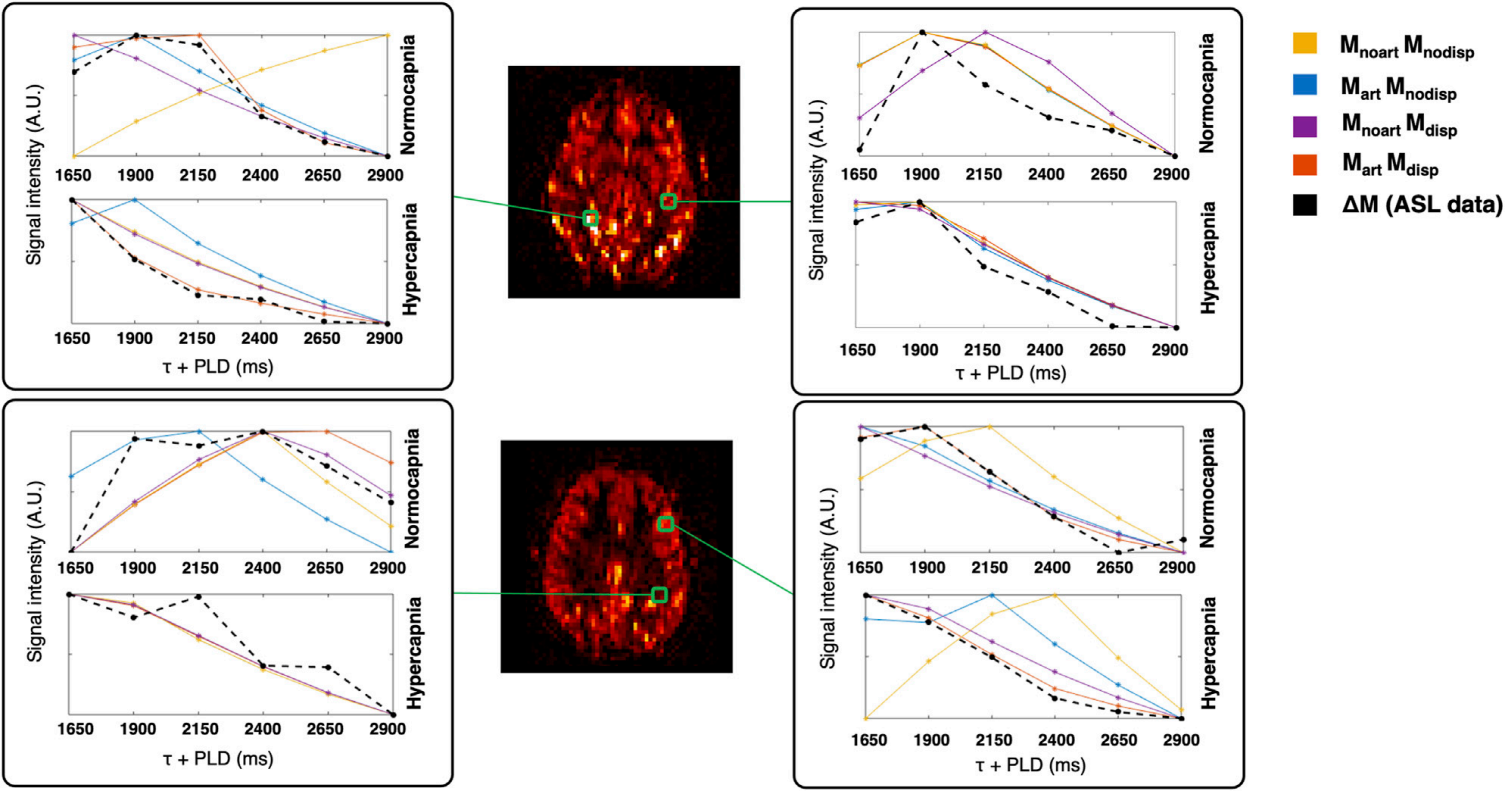
Illustrative ΔM maps (two different axial slices, normocapnia, signal intensity in arbitrary units). Four different voxels were selected (green) and the corresponding voxelwise model fittings are displayed in different colours for each condition.

Figure 3 shows two illustrative ΔM maps. Several voxels were selected and their corresponding four model fittings for each one of the conditions can be seen. This figure further highlights the different dynamics between conditions and the impact of the different modelling strategies.

**FIGURE 4:**
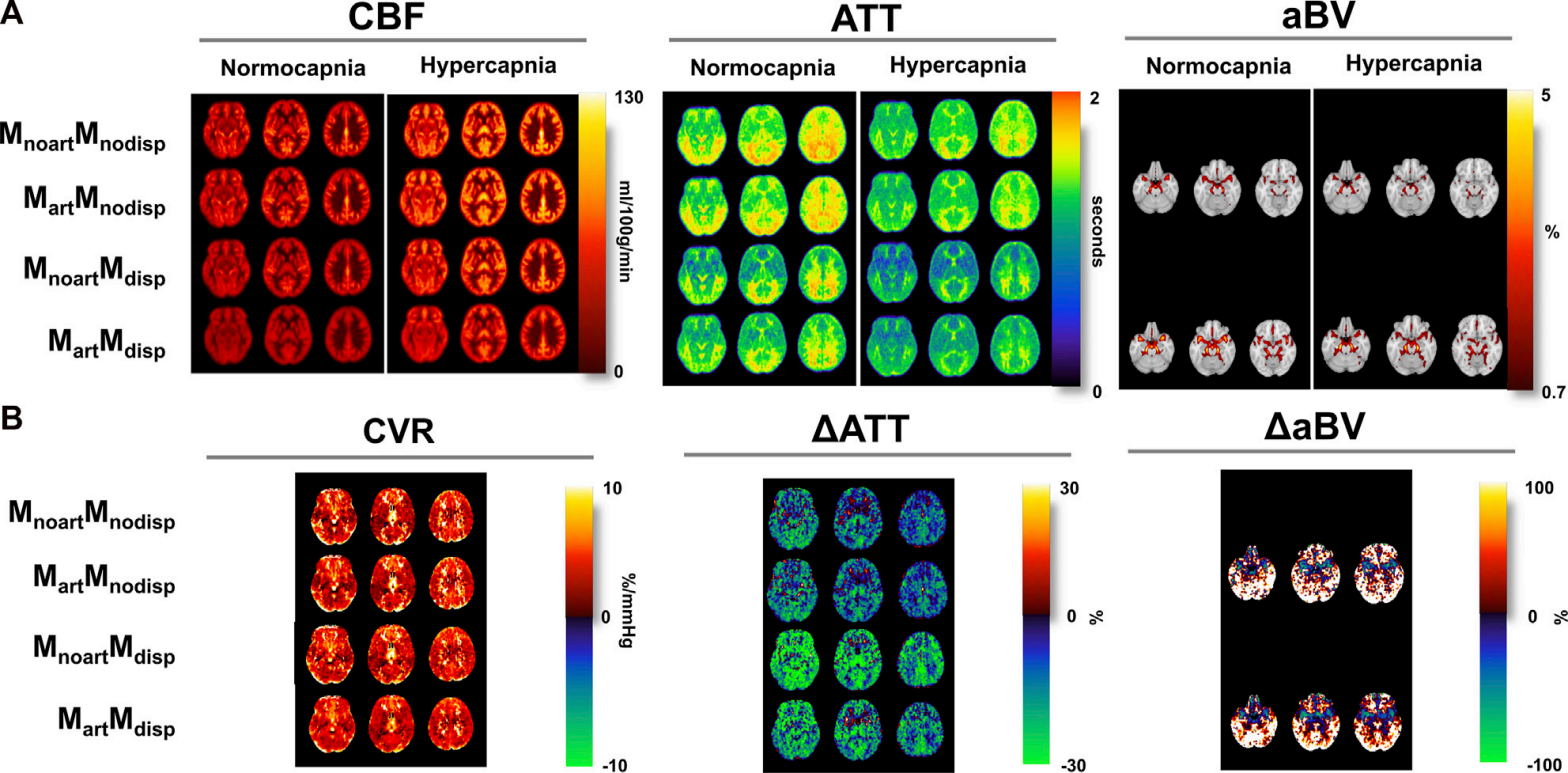
**(A)** Orthogonal representations of group average of the CBF, ATT, and aBV maps for the two conditions (normocapnia and hypercapnia) obtained using different modelling strategies (MNI space). **(B)** CVR, and difference ATT (ΔATT) and aBV (ΔaBV) maps (difference between hypercapnia and normocapnia). aBV and ΔaBV maps were only obtained when using strategies where the macrovascular component was modelled (M_art_).

Figure 4 displays the CBF, ATT, and aBV maps averaged across subjects, obtained using the four different modelling strategies, and during the two conditions, as well as the corresponding differences in CBF (CVR), ATT (ΔATT) and aBV (ΔaBV) across conditions maps. Representations of illustrative individual CBF, ATT, and aBV maps can be found in the Supplementary Material.

Several differences can be observed across the parameter maps. For instance, when comparing the conditions regardless of the model used, hypercapnia yielded maps showing higher CBF, shorter ATT in most regions, and less-defined areas of thresholded aBV (aBV with lower values).

The tested models also led to differences across the haemodynamic parameter maps. Specifically, for CBF maps, brighter areas localised around the major arteries can be seen in models that do not account for the macrovascular component or dispersion effects. Models that account for dispersion effects also tend to yield lower CBF values across the brain, and the model that includes both the macrovascular component and dispersion effects produced more homogeneous CBF maps in both conditions, and in the CVR maps (CBF difference maps). The CVR maps also show high and unrealistic values in some regions including brain edges and WM regions, but this might be due to low SNR resulting in poor fitting (Figure 3), that is amplified when computing CVR due to the normalization step. These erroneous high CVR regions appear to be less frequent when including the arterial and dispersion components into the model.

Regarding ATT, the occipital and superior areas tend to display higher values in all models/conditions tested in comparison with other brain regions. When using models that include dispersion effects, the corresponding ATT maps show higher contrast between specific regions such as GM/WM as well as cortical/sub-cortical areas (e.g., lower ATT in the putamen and globus pallidus). The differences between conditions, displayed by the ΔATT, are higher when using the dispersion models. ΔATT is also higher during hypercapnia (positive values), in some specific frontal areas.

Including dispersion in the modelling also leads to higher aBV areas, in particular, in lateral regions further downstream. The differences between conditions are higher if including dispersion effects into the model.

**FIGURE 5:**
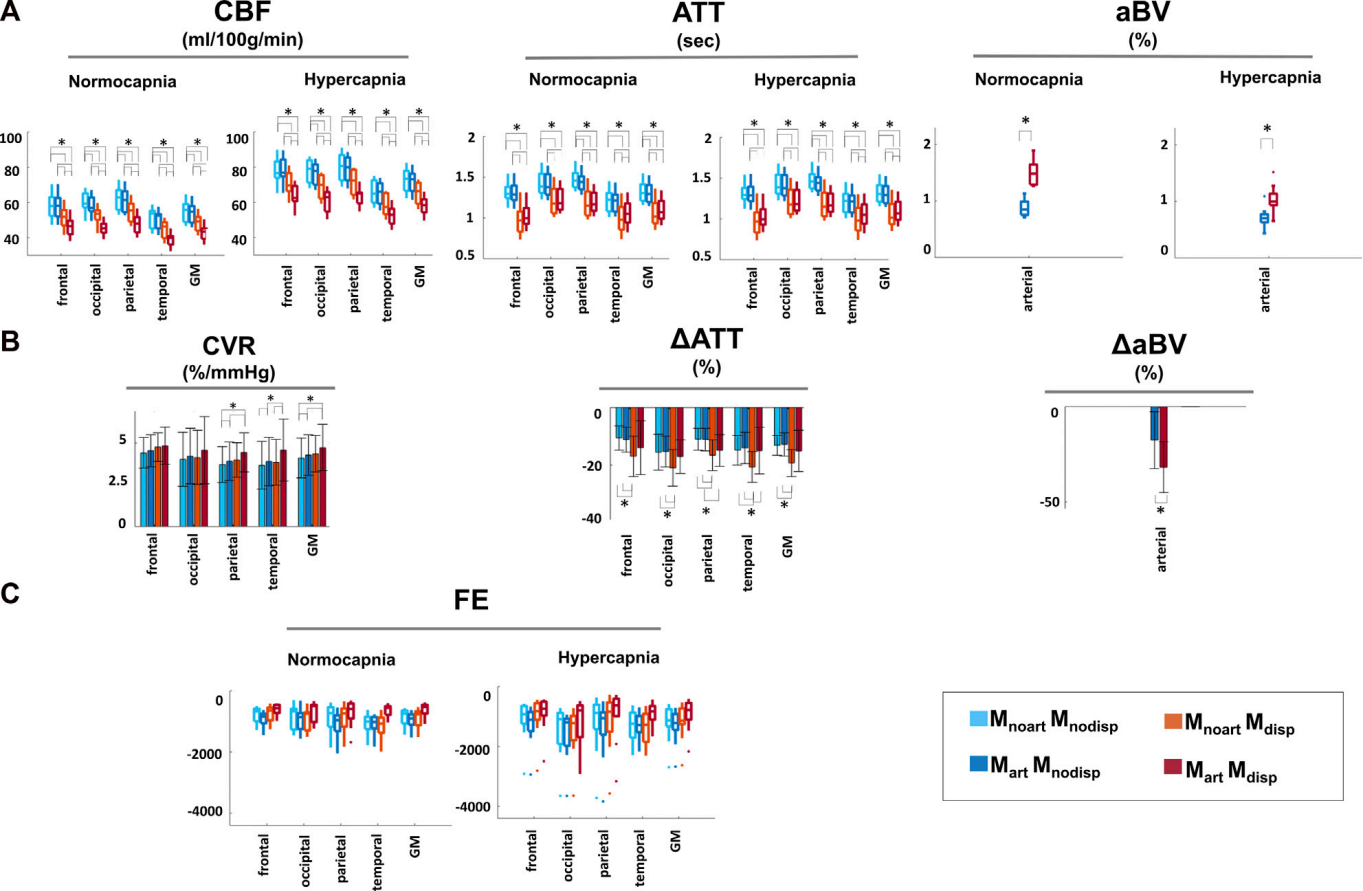
Regional analysis of: **(A)** CBF, ATT, and aBV with the different modelling strategies and conditions. **(B)** Percentage change of CBF, ATT and aBV normocapnia vs. hypercapnia. CVR corresponds to CBF change normalized against the change in the individual PETCO_2_ level (%/mmHg). **(C)** Regional FE for each of the different modelling strategies and conditions Statistically significant results (*p* < 0.05) are highlighted with *.

Figure 5 shows the results of the regional analysis for CBF, ATT, aBV, and FE for each model and condition and the corresponding regional change in CBF (CVR), ΔATT and ΔaBV values between conditions for each model. Statistically significant main effects in CBF were observed for all factors tested (condition, model, and regions), as well as for the interactions between these (all *p* ≤ 0.01). Post-hoc comparisons of CBF across models yielded significant differences (*p* < 0.05), except for the inclusion of an arterial component in models that do not account for dispersion (M_noart_M_nodisp_ and M_art_M_nodisp;_ blue bar plots) in specific regions/conditions (GM changes across the different models in relation to M_noart_M_nodisp_: ∼0.6–22%). Significant main effects in ATT measures were also observed for all factors tested and pairwise interactions (*p* < 0.05). Pairwise comparisons of ATT across models and conditions yielded significant differences (*p* < 0.05) except for M_noart_M_nodisp_ and M_art_M_nodisp_ (blue bar plots) and for M_noart_M_disp_ and M_art_M_disp_ (red/orange bar plots) in specific regions/conditions (GM changes across the different models in relation to M_noart_M_nodisp_: ∼0.6–19%). For average aBV, significant main effects were obtained for condition and model and their interaction (*p* < 0.01). Pairwise comparisons between the two models were all significant in both conditions (in relation to M_art_M_nodisp_ ∼ 74 and 44% for normocapnia and hypercapnia, respectively). Statistically significant main effects were observed in FE for all factors/interactions (*p* < 0.05), except for factor condition (*p* = 0.077). Regardless of the condition and region tested, significant differences were obtained when comparing models except for M_noart_M_nodisp_ and M_noart_M_disp_. The model including dispersion and macrovascular components consistently yields FE values closer to zero across models, i.e., better model fit.

Focusing on differences between conditions, there was a significant main effect for the factor model (*p* < 0.01), but not for factors region/interaction. While CBF changes (CVR) were significantly higher with the model that includes dispersion and the arterial component in specific areas including GM, for ATT the highest changes were obtained when modelling only dispersion, thus there seems to be an interaction between these two modelling options that depends on the parameter estimated.

## 4 Discussion

In this work, we analysed pCASL data during two conditions (normocapnia and hypercapnia) and using different kinetic models. Our results highlight the different CBF spatiotemporal dynamics across conditions: hypercapnia led to a significant increase in CBF and ATT, whilst aBV decreased significantly. Moreover, parameter quantification was also significantly affected by model selection. Incorporation of dispersion effects yielded a significant decrease in CBF and ATT and aBV increased in both conditions. Overall, the extended model that includes dispersion effects and the macrovascular component provides the best fit to both datasets (in terms of FE).

### 4.1 Data acquisition and gas challenge

Hypercapnia was attained through a respiratory challenge that increased the CO_2_ content of arterial blood by applying a prospective end-tidal targeting and maintenance method using a computer-controlled gas blender. This approach has been shown to be a robust and reliable way to prospectively induce changes in the arterial blood CO_2_ content, whilst targeting and maintaining stable O_2_ levels (Fierstra et al., 2013). Although the target value of an increase of 10 mmHg to each subject’s baseline PETCO_2_ was not attained, all subjects completed the gas challenge and experienced a similar PETCO_2_ change within a standard deviation of 0.8 mmHg (Table 2). The two conditions yielded statistically significantly different PETCO_2_ average values (*p* < 0.01) (Table 2).

### 4.2 Impact of different conditions on CBF dynamics quantification

Although literature on the effect of hypercapnia using multiple-PLD ASL is still limited, our results are in agreement with other reports that confirm a change in CBF dynamics with an increase in amplitude and a faster response due to hypercapnia (Donahue et al., 2016). These different dynamics can be perceived even before model fitting, as illustrated by the ΔM/M_0_ maps and curves of the two conditions (Figure 2), and during model fitting (Figure 3).

Our quantitative changes of CBF (∼33%) and ATT (∼15%) are slightly higher than the ones previously reported, although these differences might be partially explained by the different stimuli and processing analyses used. Donahue et al. reported reductions in ATT in the order of 4.6%–7.7% and a CBF increase of 8.2%–27.8% when using a pCASL sequence with a fixed-inspired challenge (inspired fraction of 5%) (Donahue et al., 2016), while Ho and others observed a GM CBF increase of around 21% and an ATT decrease in the GM of approximately 5% when using an ASL-QUASAR strategy in combination with an increase in PETCO_2_ content by a third of the subject’s baseline (∼14 mm Hg) (Ho et al., 2011).

The spatiotemporal patterns in the dynamics during hypercapnia are also in line with previous reports (MacIntosh et al., 2010; Donahue et al., 2014). While the impact of hypercapnia on CBF appears to be statistically significant across the brain, for ATT, hypercapnia appears to mainly affect posterior and lateral regions, without statistically significant changes in frontal regions. Other ASL studies have also indicated prolonged ATT in border zone regions between the major cerebral artery territories (Petersen et al., 2010), highlighting the dependence of these areas on the individual vascular architecture and geometry (Wong et al., 1997).

The arterial component during hypercapnia also seems to be lower and less defined than during normocapnia. This can be explained by the increased flow velocity in arteries resulting in the tagged blood arriving earlier in combination with the non-optimal ASL sampling scheme in terms of PLDs during hypercapnia. Ho et al. also investigated the impact of hypercapnia on the arterial component, observing dissociations in the dynamics between large vessels and GM. In particular, there were significant changes in the aBV of the larger arteries (∼11%) and this difference was approximately half of the CBF increase (Ho et al., 2011).

### 4.3 Impact of model strategies on CBF dynamics quantification during different conditions

To our knowledge, this is the first study to assess the individual and combined impact of modelling dispersion and macrovascular components in multiple-PLD ASL haemodynamic parameter quantification during hypercapnia. Our results highlight the influence of modelling strategies on parameter quantification, even when not taking different conditions into account, which is in line with previous reports. In particular, the inclusion of dispersion into the model yielded lower CBF, ATT, and higher aBV, while more arterial signal was fitted, particularly in arteries further downstream. This can be seen visually in the parameter maps of Figure 4, where including dispersion led to less variable CBF maps in both conditions, while correcting for macrovascular areas. In fact, there seems to be an interaction between these two modelling aspects, with no significant difference in parameter quantification when introducing a macrovascular component in most regions/conditions if dispersion is not included. If dispersion is already included, adding the macrovascular component leads to significant differences in parameter quantification, with an overall decrease in CBF and an increase in ATT, likely due to better tissue and arterial signal separation. This is confirmed by the signal changes when comparing the two conditions, as CBF changes between conditions (CVR) are higher when including the arterial component, while for ATT are higher without including this component.

The impact of including dispersion also appears to be region dependent. For instance, the contrast between WM in relation to cortical GM seems to be higher when introducing dispersion into the model which is in line with the expectation that the impact of dispersion modelling will be more pronounced deeper into the vascular tree, hence, introducing this component might improve modelling and ATT quantification across WM (Figure 4). Subcortical GM areas also tend to display a different profile ratio in relation to cortical GM when including dispersion, where ATT values in subcortical areas are lower than in cortical areas.

Similarly, the impact of modelling strategies in parameter changes due to hypercapnia are also model and region dependent. For example, while the CBF changes when using M_noart_M_disp_ or M_art_M_disp_ in the frontal area are not significantly different, for other areas such as temporal or occipital regions, these two models yield significant differences. These regional differences and model/parameter dependencies might be due to the distinctive dynamics of blood coming from different main feeding arteries and their interaction with model fitting. Including both the dispersion and the macrovascular component seems to incorporate/alleviate some of these vascular differences.

The arterial component is also better distinguished when adding dispersion into the model, particularly in regions further downstream, which is in line with the assumption of a higher impact of dispersion modelling in deeper areas within the vascular tree. This effect seems to be higher during normocapnia, as the increase in blood velocity due to hypercapnia possibly makes the multi-PLD sampling scheme not optimised for this condition, as the earliest PLD might be too long to accurately detect the macrovascular contribution.

Moreover, our FE values also support the application of the model that includes dispersion and the macrovascular component, since this combination yielded the FE values closest to zero across models and conditions (i.e., better fit to the data). Although, the CVR maps obtained seem to be similar across the models tested, the model that includes both dispersion and the macrovascular component appears to create maps with lower variability, also yielding significantly higher regional values of CVR when compared with the other models.

Most of these parameter quantification differences due to modelling strategies are in line with previous reports on normocapnia (Zhang et al., 2021; van der Plas et al., 2022). A recent study comparing different ASL sequence optimization strategies in normocapnia, showed that sequences optimized for both ATT and CBF estimation are sensitive to macrovascular signal and that including dispersion effects and the macrovascular component leads to significant decreases in CBF and ATT estimation errors (Zhang et al., 2021).

As seen in other ASL studies, our CVR maps also do not display the clear GM/WM contrast commonly seen in BOLD CVR maps. Some studies argue that the tissue difference in BOLD CVR maps might be the result of the complex interaction between several physiological parameters, and the lack of differences in ASL might be reasonable as both baseline CBF and CBF induced changes by hypercapnia might be lower in WM (Taneja et al., 2019).

### 4.4 Limitations and future work

Although differences between parameters across conditions and models were observed and quantified, a major limitation of this work is the lack of a gold standard measure to compare our results with. In the future, a comparison with other methodologies such as PET imaging should be performed (Zhao et al., 2021). Additionally, although hypercapnic stimuli are commonly used for CVR mapping, some pathological conditions might mimic the spatiotemporal changes in CBF dynamics seen with hypercapnia (for example, if the basal vascular tone is altered or arterial blood velocities are reduced (Bright et al., 2011)). Another major limitation of this work is the small sample size (*n* = 10). Additionally, some of the analysis options used in this work were dependent on the ASL acquisition parameters/strategies. Given the faster flow in large arteries and the earlier arrival times of labelled blood during hypercapnia, it might be important to investigate the impact of optimising the ASL sampling scheme when acquiring data during this condition (Woods et al., 2019). Regarding the macrovascular signal, alternative strategies can be used to remove this component during acquisition, such as including the use of flow crusher gradients. However, this approach has been discouraged (Alsop et al., 2015) and by removing this component, important clinical information might be overlooked. Another aspect that warrants further investigation is the large variability in anatomical features across and within individuals (different vascular architecture and territories) and its impact on ASL modelling, particularly in dispersion effects. Including a combination of functional and structural information might be beneficial to better model the ASL signal by taking these vascular differences into account (Li et al., 2018). Conflicting results have been reported when comparing the impact of blood flow velocity on ASL imaging (Aslan et al., 2010; Heijtel et al., 2014; Dolui et al., 2016). Although these differences can impact CVR results, these will not affect our conclusions regarding the impact of the different model strategies within each conditions, as the effect of the different labelling efficiencies will be the same across the models tested.

Importantly, our observations regarding the impact of the different models on parameter quantification can also have implications when evaluating CBF in pathologies. In several conditions, including steno-occlusive diseases, brain tumors or arteriovenous malformations (Amemiya et al., 2022; Hirschler et al., 2023), the dynamics of blood vessels and flow are known to be altered and these will likely depend on the degree of disease severity and underlying etiology. In those cases, using multi-PLD ASL strategies in combination with modelling strategies that take into account these differences, including models with dispersion and macrovascular component, might be beneficial for a more accurate estimation of CBF parameters. Further work on ASL modelling strategies should focus on translating/validating these findings in clinical applications.

## 5 Conclusion

This work highlights the significance of acquiring ASL data using a multiple-PLD approach to allow a larger flexibility in ASL parameter estimation, and the critical aspect of making anatomically and physiologically valid assumptions when modelling ASL data. Here we recommend the use of extended models that include the macrovascular component and dispersion effects when modelling multiple-PLD pCASL data. This is of particular importance when imaging abnormal states such as increased or decreased global CBF as induced by respiratory challenges or vasoactive substances, or in subjects with pathologies that may impact their cerebral perfusion.

## Data Availability

The datasets presented in this study can be found in online repositories. The names of the repository/repositories and accession number (s) can be found below: Oxford Research Archive Repository, https://doi.org/10.5287/bodleian:Xk48adQAO.

## References

[B1] Alsop D. C. Detre J. A. Golay X. Günther M. Hendrikse J. Hernandez-Garcia L. (2015). Recommended implementation of arterial spin-labeled perfusion MRI for clinical applications: A consensus of the ismrm perfusion study group and the European consortium for ASL in dementia. Magn. Reson. Med. 73, 102–116. 10.1002/mrm.25197 24715426 PMC4190138

[B2] Amemiya S. Takao H. Watanabe Y. Takei N. Ueyama T. Kato S. (2022). Reliability and sensitivity to longitudinal CBF changes in steno-occlusive diseases: ASL versus 123I-IMP-SPECT. J. Magn. Reson. Imaging 55, 1723–1732. 10.1002/JMRI.27996 34780101

[B3] Andersson J. Jenkinson M. Smith S. (2007). Non-linear registration aka Spatial normalisation. United Kingdom: FMRIB Technical Report TR07 JA2.

[B4] Aslan S. Xu F. Wang P. L. Uh J. Yezhuvath U. S. Van Osch M. (2010). Estimation of labeling efficiency in pseudocontinuous arterial spin labeling. Magn. Reson. Med. 63, 765–771. 10.1002/mrm.22245 20187183 PMC2922009

[B5] Blockley N. Harkin J. Stone A. Bulte D. (2016). Data acquired to investigate new approaches to cerebrovascular reactivity mapping using MRI. Oxford: ORA - Oxford Univ. Res. Arch. Available at: https://ora.ox.ac.uk/objects/uuid:1801af1b-4872-40da-92df-a9ecbe839e4f (Accessed 5 5, 22).

[B6] Blockley N. P. Harkin J. W. Bulte D. P. (2017). Rapid cerebrovascular reactivity mapping: Enabling vascular reactivity information to be routinely acquired. Neuroimage 159, 214–223. 10.1016/J.NEUROIMAGE.2017.07.048 28756241

[B7] Bright M. G. Donahue M. J. Duyn J. H. Jezzard P. Bulte D. P. (2011). The effect of basal vasodilation on hypercapnic and hypocapnic reactivity measured using magnetic resonance imaging. J. Cereb. Blood Flow. Metab. 31, 426–438. 10.1038/jcbfm.2010.187 20959855 PMC3049535

[B8] Buxton R. B. Frank L. R. Wong E. C. Siewert B. Warach S. Edelman R. R. (1998). A general kinetic model for quantitative perfusion imaging with arterial spin labeling. Magn. Reson. Med. 40, 383–396. 10.1002/mrm.1910400308 9727941

[B9] Catchlove S. J. Pipingas A. Hughes M. E. Macpherson H. (2018). Magnetic resonance imaging for assessment of cerebrovascular reactivity and its relationship to cognition: A systematic review. BMC Neurosci. 19, 21–15. 10.1186/s12868-018-0421-4 29649969 PMC5898077

[B10] Chappell M. A. Groves A. R. Whitcher B. Woolrich M. W. (2009). Variational bayesian inference for a nonlinear forward model. IEEE Trans. Signal Process. 57, 223–236. 10.1109/TSP.2008.2005752

[B11] Chappell M. A. MacIntosh B. J. Donahue M. J. Günther M. Jezzard P. Woolrich M. W. (2010). Separation of macrovascular signal in multi-inversion time arterial spin labelling MRI. Magn. Reson. Med. 63, 1357–1365. 10.1002/mrm.22320 20432306

[B12] Chappell M. A. Woolrich M. W. Kazan S. Jezzard P. Payne S. J. MacIntosh B. J. (2013). Modeling dispersion in arterial spin labeling: Validation using dynamic angiographic measurements. Magn. Reson. Med. 69, 563–570. 10.1002/mrm.24260 22489046

[B13] Chen J. J. (2018). Cerebrovascular-reactivity mapping using MRI: Considerations for Alzheimer’s disease. Front. Aging Neurosci. 11, 170–179. 10.3389/fnagi.2018.00170 PMC5996106 29922153

[B14] De Vis J. B. Bhogal A. A. Hendrikse J. Petersen E. T. Siero J. C. W. (2018). Effect sizes of BOLD CVR, resting-state signal fluctuations and time delay measures for the assessment of hemodynamic impairment in carotid occlusion patients. Neuroimage 179, 530–539. 10.1016/j.neuroimage.2018.06.017 29913284 PMC6057274

[B15] Dolui S. Wang Z. Wang D. J. J. Mattay R. Finkel M. Elliott M. (2016). Comparison of non-invasive MRI measurements of cerebral blood flow in a large multisite cohort. J. Cereb. Blood Flow. Metab. 36, 1244–1256. 10.1177/0271678X16646124 27142868 PMC4929707

[B16] Donahue M. J. Faraco C. C. Strother M. K. Chappell M. A. Rane S. Dethrage L. M. (2014). Bolus arrival time and cerebral blood flow responses to hypercarbia. J. Cereb. Blood Flow. Metab. 34, 1243–1252. 10.1038/jcbfm.2014.81 24780904 PMC4083394

[B17] Donahue M. J. Strother M. K. Lindsey K. P. Hocke L. M. Tong Y. Frederick B. deB. (2016). Time delay processing of hypercapnic fMRI allows quantitative parameterization of cerebrovascular reactivity and blood flow delays. J. Cereb. Blood Flow. Metab. 36, 1767–1779. 10.1177/0271678X15608643 26661192 PMC5076782

[B18] Fierstra J. Sobczyk O. Battisti-Charbonney A. Mandell D. M. Poublanc J. Crawley A. P. (2013). Measuring cerebrovascular reactivity: What stimulus to use? J. Physiol. 591, 5809–5821. 10.1113/jphysiol.2013.259150 24081155 PMC3872753

[B19] Gallichan D. Jezzard P. (2008). Modeling the effects of dispersion and pulsatility of blood flow in pulsed arterial spin labeling. Magn. Reson. Med. 60, 53–63. 10.1002/mrm.21654 18581416

[B20] Haller S. Zaharchuk G. Thomas D. L. Lovblad K. O. Barkhof F. Golay X. (2016). Arterial spin labeling perfusion of the brain: Emerging clinical applications. Radiology 281, 337–356. 10.1148/RADIOL.2016150789 27755938

[B21] Heijtel D. F. R. Mutsaerts H. J. M. M. Bakker E. Schober P. Stevens M. F. Petersen E. T. (2014). Accuracy and precision of pseudo-continuous arterial spin labeling perfusion during baseline and hypercapnia: A head-to-head comparison with ¹⁵O H₂O positron emission tomography. Neuroimage 92, 182–192. 10.1016/j.neuroimage.2014.02.011 24531046

[B22] Hirschler L. Sollmann N. Schmitz-Abecassis B. Pinto J. Arzanforoosh F. Barkhof F. (2023). Advanced MR techniques for preoperative glioma characterization: Part 1. J. Magn. Reson. Imaging 2023, 28662. 10.1002/JMRI.28662 PMC10946498 36866773

[B23] Ho Y. C. L. Petersen E. T. Zimine I. Golay X. (2011). Similarities and differences in arterial responses to hypercapnia and visual stimulation. J. Cereb. Blood Flow. Metab. 31, 560–571. 10.1038/JCBFM.2010.126 20700127 PMC3049511

[B24] Jenkinson M. Bannister P. Brady M. Smith S. (2002). Improved optimization for the robust and accurate linear registration and motion correction of brain images. Neuroimage 17, 825–841. 10.1016/s1053-8119(02)91132-8 12377157

[B25] Jenkinson M. Beckmann C. F. Behrens T. E. J. Woolrich M. W. Smith S. M. (2012). FSL. Neuroimage 62, 782–790. 10.1016/j.neuroimage.2011.09.015 21979382

[B26] Kazan S. M. Chappell M. A. Payne S. J. (2009). Modeling the effects of flow dispersion in arterial spin labeling. IEEE Trans. Biomed. Eng. 56, 1635–1643. 10.1109/TBME.2009.2016977 19307163

[B27] Li Y. Mao D. Li Z. Schär M. Pillai J. J. Pipe J. G. (2018). Cardiac-triggered pseudo-continuous arterial-spin-labeling: A cost-effective scheme to further enhance the reliability of arterial-spin-labeling MRI. Magn. Reson. Med. 80, 969–975. 10.1002/MRM.27090 29369422 PMC5980664

[B28] Lindner T. Bolar D. S. Achten E. Barkhof F. Bastos-Leite A. J. Detre J. A. (2023). Current state and guidance on arterial spin labeling perfusion MRI in clinical neuroimaging. Magn. Reson. Med. 89, 2024–2047. 10.1002/MRM.29572 36695294 PMC10914350

[B29] MacIntosh B. J. Lindsay A. C. Kylintireas I. Kuker W. Gunther M. Robson M. D. (2010). Multiple inflow pulsed arterial spin-labeling reveals delays in the arterial arrival time in minor stroke and transient ischemic attack. Am. J. Neuroradiol. 31, 1892–1894. 10.3174/ajnr.A2008 20110375 PMC7964001

[B30] Mandell D. M. Han J. S. Poublanc J. Crawley A. P. Kassner A. Fisher J. A. (2008). Selective reduction of blood flow to white matter during hypercapnia corresponds with leukoaraiosis. Stroke 39, 1993–1998. 10.1161/STROKEAHA.107.501692 18451357

[B31] Mazziotta J. Toga A. Evans A. Fox P. Lancaster J. Zilles K. (2001). A probabilistic atlas and reference system for the human brain: International Consortium for Brain Mapping (ICBM). Philos. Trans. R. Soc. B Biol. Sci. 356, 1293–1322. 10.1098/rstb.2001.0915 PMC1088516 11545704

[B32] Moreton F. C. Dani K. A. Goutcher C. O’Hare K. Muir K. W. (2016). Respiratory challenge MRI: Practical aspects. NeuroImage Clin. 11, 667–677. 10.1016/j.nicl.2016.05.003 27330967 PMC4901170

[B33] Okell T. W. Chappell M. A. Kelly M. E. Jezzard P. (2013). Cerebral blood flow quantification using vessel-encoded arterial spin labeling. J. Cereb. Blood Flow. Metab. 33, 1716–1724. 10.1038/jcbfm.2013.129 23921895 PMC3824178

[B34] Petersen E. T. Mouridsen K. Golay X. (2010). The QUASAR reproducibility study, Part II: Results from a multi-center Arterial Spin Labeling test-retest study. Neuroimage 49, 104–113. 10.1016/j.neuroimage.2009.07.068 19660557 PMC2768325

[B35] Pinto J. Bright M. G. Bulte D. P. Figueiredo P. (2021). Cerebrovascular reactivity mapping without gas challenges: A methodological guide. Front. Physiol. 11, 608475. 10.3389/fphys.2020.608475 33536935 PMC7848198

[B36] Pinto J. Chappell M. A. Okell T. W. Mezue M. Segerdahl A. R. Tracey I. (2020). Calibration of arterial spin labeling data—Potential pitfalls in post‐processing. Magn. Reson. Med. 83, 1222–1234. 10.1002/mrm.28000 31605558 PMC6972489

[B37] Slessarev M. Han J. Mardimae A. Prisman E. Preiss D. Volgyesi G. (2007). Prospective targeting and control of end-tidal CO2 and O2 concentrations. J. Physiol. 581, 1207–1219. 10.1113/jphysiol.2007.129395 17446225 PMC2170842

[B38] Smith S. (2002). Fast robust automated brain extraction. Hum. Brain Mapp. 17, 143–155. 10.1002/hbm.10062 12391568 PMC6871816

[B39] Sobczyk O. Battisti-Charbonney A. Poublanc J. Crawley A. P. Sam K. Fierstra J. (2015). Assessing cerebrovascular reactivity abnormality by comparison to a reference atlas. J. Cereb. Blood Flow. Metab. 35, 213–220. 10.1038/JCBFM.2014.184 25388679 PMC4426737

[B40] Taneja K. Lu H. Welch B. G. Thomas B. P. Pinho M. Lin D. (2019). Evaluation of cerebrovascular reserve in patients with cerebrovascular diseases using resting-state MRI: A feasibility study. Magn. Reson. Imaging 59, 46–52. 10.1016/j.mri.2019.03.003 30849484 PMC6494444

[B41] van der Plas M. C. E. Craig M. Schmid S. Chappell M. A. van Osch M. J. P. (2022). Validation of the estimation of the macrovascular contribution in multi-timepoint arterial spin labeling MRI using a 2-component kinetic model. Magn. Reson. Med. 87, 85–101. 10.1002/MRM.28960 34390279 PMC10138741

[B42] Wong E. C. Buxton R. B. Frank L. R. (1997). Implementation of quantitative perfusion imaging techniques for functional brain mapping using pulsed arterial spin labeling. NMR Biomed. 10, 237–249. 10.1002/(sici)1099-1492(199706/08)10:4/5<237:aid-nbm475>3.0.co;2-x 9430354

[B43] Woods J. G. Chappell M. A. Okell T. W. (2019). A general framework for optimizing arterial spin labeling MRI experiments. Magn. Reson. Med. 81, 2474–2488. 10.1002/MRM.27580 30588656 PMC6492260

[B44] Wu W. C. Mazaheri Y. Wong E. C. (2007). The effects of flow dispersion and cardiac pulsation in arterial spin labeling. IEEE Trans. Med. Imaging 26, 84–92. 10.1109/TMI.2006.886807 17243587

[B45] Zhang L. X. Woods J. G. Okell T. W. Chappell M. A. (2021). Examination of optimized protocols for pCASL: Sensitivity to macrovascular contamination, flow dispersion, and prolonged arterial transit time. Magn. Reson. Med. 86, 2208–2219. 10.1002/MRM.28839 34009682 PMC8581991

[B46] Zhang Y. Brady M. Smith S. (2001). Segmentation of brain MR images through a hidden Markov random field model and the expectation-maximization algorithm. IEEE Trans. Med. Imaging 20, 45–57. 10.1109/42.906424 11293691

[B47] Zhao M. Y. Fan A. P. Chen D. Y. T. Sokolska M. J. Guo J. Ishii Y. (2021). Cerebrovascular reactivity measurements using simultaneous 15 O-water PET and ASL MRI: Impacts of arterial transit time, labeling efficiency, and hematocrit. Neuroimage 233, 117955. 10.1016/J.NEUROIMAGE.2021.117955 33716155 PMC8272558

