# Supplementary material for: Modelling spatiotemporal dynamics of cerebral blood flow using multiple-timepoint arterial spin labelling MRI
Source: Front Physiol. 2023 May 26;14:1142359. doi: 10.3389/fphys.2023.1142359 (PMC10250662; doi:10.3389/fphys.2023.1142359)
Supplement: Supplementary file 1 [file DataSheet1.docx]

**Supplementary Material**


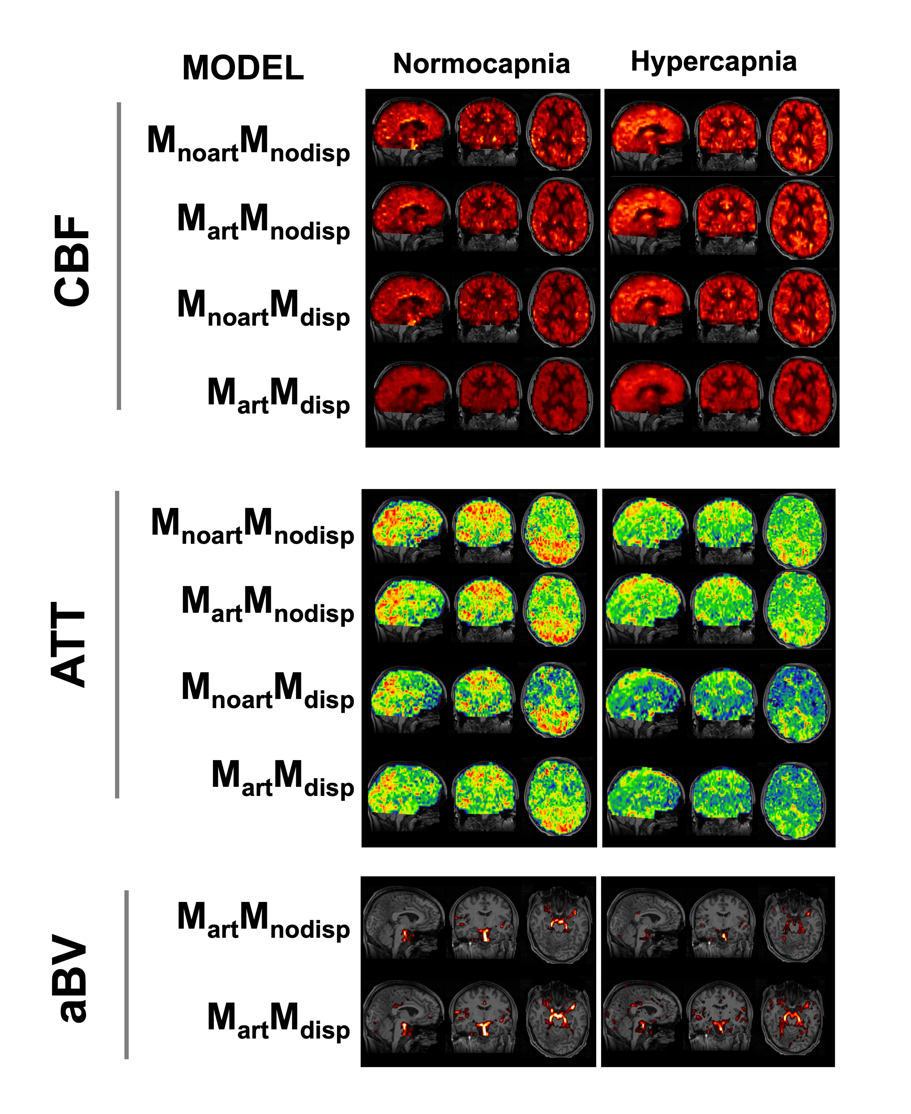


Figure S1. Orthogonal representations of illustrative individual CBF, ATT, and aBV maps for the two conditions (normocapnia and hypercapnia), obtained using different modelling strategies (all in structural space). aBV maps are only obtained when using strategies where the macrovascular component is modelled (M_art_).
